# Mitochondria transfer in glioblastoma

**DOI:** 10.1093/neuonc/noag038

**Published:** 2026-02-23

**Authors:** Simon Storevik, Mina Thue Augustsson, Shannon Moreino, Hanjo Köppe, Dionysios C Watson, Defne Bayik, Carolina De La Pena Fernandez, Amanda M Serapiglia, Nikhil Panicker, Justin Lathia, Hrvoje Miletic

**Affiliations:** Department of Neurology, Haukeland University Hospital, Bergen, Norway; Department of Biomedicine, University of Bergen, Bergen, Norway; Department of Biomedicine, University of Bergen, Bergen, Norway; Institute of Pharmacology, University of Greifswald, Greifswald, Germany; Department of Neurosurgery, University Medicine Greifswald, Greifswald, Germany; Medical Oncology, Miller School of Medicine, Miami, USA; Molecular and Cellular Pharmacology, Miller School of Medicine, Miami, USA; Medical Oncology, Miller School of Medicine, Miami, USA; Department of Neurosciences, Case Western Reserve University School of Medicine, Cleveland, USA; Department of Neurosciences, Cleveland Clinic Research, Cleveland, USA; Department of Neurosciences, Cleveland Clinic Research, Cleveland, USA; Department of Clinical Medicine, University of Copenhagen, Copenhagen, Denmark; Department of Cancer Sciences, Cleveland Clinic Research, Cleveland, USA; Department of Biomedicine, University of Bergen, Bergen, Norway; Department of Pathology, Haukeland University Hospital, Bergen, Norway

## Abstract

Glioblastoma (GBM) is a highly aggressive and metabolically adaptable brain tumor characterized by profound cellular heterogeneity and therapy resistance. Recent research has uncovered the phenomenon of horizontal mitochondrial transfer (HMT) between GBM cells and their microenvironment, particularly astrocytes, which contributes to tumor progression, metabolic reprogramming, and treatment resistance. This review summarises current knowledge on mitochondrial exchange in GBM via tunneling nanotubes (TNTs), tumor microtubes (TMs) and potentially via extracellular vesicles (EVs). It also explores the functional consequences of HMT, including enhanced oxidative phosphorylation (OXPHOS), increased tumorigenicity, and altered therapeutic responses. This review highlights the need for further investigation into the molecular drivers and context-specific outcomes of mitochondrial transfer in GBM, with implications for novel therapeutic strategies.

Key Points

- GBM cells exchange mitochondria through TMs and TNTs, and potentially via EVs and soluble routes.
- Astrocytes are major donors of mitochondria to GBM cells.
- HMT promotes proliferation, self-renewal, OXPHOS and treatment resistance.

Glioblastoma (GBM) is the most common primary tumour of the central nervous system (CNS) in adults.^1^ Only limited therapeutic or prognostic gains have been made in recent years,^2^ which is reflected in a median survival around 14 to 17 months after diagnosis and a 5 year survival of 3-5%.^1^ Therapy resistance remains a significant obstacle,^3^ partly explained by a continuous tumor evolution leading to comprehensive cellular diversity and subpopulations with distinct characteristics.^4^ GBM is known as one of the most heterogenous cancer types,^4^ and an inverse relationship between the degree of heterogeneity and the effectiveness of therapy has been reported.^5^

The invasive growth pattern of GBM is a significant element in disease recurrence and adverse prognosis.^6^ GBM invade as single cells and migrate deeper compared to other brain tumor types,^7^ typically along pre-existing brain structures.^6^ This process demands a high degree of metabolic adaptability to adjust to new microenvironments where accessibility to oxygen and nutrients differ.^8^ GBM relies on several metabolic pathways,^9^^,^^10^ that can vary based on how the tumor interacts with its surrounding microenvironment.^6^ GBM exhibit aberrant consumption of glucose as well as other biomolecules like glutamine, fatty acids and nucleotides.^9^^,^^10^ The different approaches used by GBM subpopulations to harvest energy is evidence of the underlying metabolic heterogeneity, and these strategies include oxidative phosphorylation (OXPHOS),^9–13^ aerobic glycolysis^10^ and fatty acid oxidation.^10^

In addition to direct metabolic regulation by the metabolite composition of the TME, emerging evidence supports a strong role for GBM reprogramming via intercellular exchange of mitochondria, the primary metabolic organelle of eukaryotic cells.^14^ This process of horizontal mitochondria transfer (HMT) involves organelle exchange either between the same type of cells (homotypic transfer) or between different cell types (heterotypic transfer).^15^ Initially, mitochondria transfer has been described between normal cells. The first report indicating intercellular mitochondria transfer was published in 2006, yet the mode of transfer as well as the question of whether whole mitochondria vs mitochondrial DNA (mtDNA) and/or other mitochondrial components were transferred remained unresolved.^16^ A decade later, HMT was demonstrated in the brain from astrocytes to neurons after stroke^17^ implicating cellular stress as a trigger for increased metabolic demands. High metabolic activity is a hallmark of cancer and mitochondria transfer from the microenvironment may contribute to metabolic adaptability of cancer cells. Initially, HMT has been studied in cancer cells depleted of mitochondrial DNA. The transfer of host mitochondria restored tumorigenic potential and respiration capacity in these cells.^18^ A very recent study further supported these findings by demonstrating mitochondria transfer to GBM cells depleted of mitochondrial DNA.^19^ The authors demonstrated that functional mitochondrial respiration was essential for in vivo tumor growth, as it supports coenzyme Q redox cycling required for de novo pyrimidine biosynthesis, which is regulated by the respiration-dependent dihydroorotate dehydrogenase (DHODH) enzyme.

The transfer of mitochondria challenges the traditional view of mitochondrial localization and behaviour,^20^^,^^21^ as new mitochondria are incorporated into a foreign mitochondrial network with bioenergetic and functional consequences.^21^ The idea of mitochondria transfer in GBM is intriguing, as GBM mitochondria are traditionally regarded as either dysfunctional^22^ or as a mixture of healthy and damaged organelles.^23^ Furthermore, recent emphasis on heterogenic metabolic strategies indicate less reliance on aerobic glycolysis than previously believed.^11^ Indeed, OXPHOS is now viewed as an important metabolic strategy, at least amongst some GBM cell types.^13^^,^^24^

In this review, we summarize the current knowledge on mechanisms of HMT in GBM and its microenvironment by focusing on major communication pathways that have been described to mediate transfer. We further depict the functional impact of HMT on tumor metabolism, tumor growth and treatment resistance. Research on mitochondria transfer in GBM is still in its infancy and future efforts are needed to delineate mechanisms of transfer including the movemement of mitochondria between communicating cells and its downstream consequences.

## Communication Pathways in GBM

HMT is dependent on specific communicaton pathways between tumor cells and its microenvironment. These pathways are in general also important for the tumorigenic process. GBM cells utilize both direct and indirect crosstalk to produce protumorigenic effects.^25^^,^^26^ Indirect avenues employ soluble elements like extracellular vesicles (EVs), growth factors and cytokines, while direct communication is mediated via cytoplasmic extensions, gap junctions (GJs) and ion channels.^25–28^

### Tunneling Nanotubes and Tumor Microtubes

Tunneling nanotubes (TNTs) were first described in 2004 based on cultures containing rat pheochromocytoma P12 cells, human embryonic kidney cells and normal rat kidney cells.^29^ These intercellular structures had a diameter of 50-200 nm, contained F-actin and were integrated in the cell membranes of the connected cells.^29^ The formation of TNTs was subsequently shown to be mediated by cytoskeletal regulators such as M-Sec and small Rho family GTPases such as CDC42 and Rac1.^30^ When connecting a large number of cells, TNTs mediated the formation of complex cellular networks.^29^ Between the interconnected cells, unidirectional travel of Lyso-tracker labelled acidic organelles was demonstrated, which was in accordance with the postulated cell membrane continuity.^29^ The discovery of motile organelles within TNTs opened new avenues for investigating mitochondrial transfer. TNTs have since been shown in a large number of cell types and are today considered a general principle of cell-to-cell communication.^30^ In 2021, TNTs were detected in GBM organoids.^31^

Another type of cytoplasmic connections are tumor microtubes (TMs). These are ultra-long cellular protrusions found specifically in GBM and isocitrate dehydrogenase (IDH) mutated astrocytoma.^32^ They were discovered in 2015 and appeared at the invasive tumor front as dynamically extending and retracting protrusions containing F-actin.^32^ Based on their unique morphological and structural features, they were deemed to be a novel subtype of membrane protrusions in GBM.^32^ TMs are wider and longer compared to TNTs, they typically have a diameter of 1-2 µm, a length of >500 µm and are connected by GJs through the protein connexin 43 (Cx43).^32^ TMs are also more stable compared to TNTs; TM lifetime is up to 200 days *in vivo* while TNTs have been observed up to 60 minutes. TMs were shown to be driven by the overexpression of the growth-associated protein 43 (GAP43),^32^ the TGF-β/TSP1 signaling axis,^33^ Tweety-Homolog 1^34^ and CHI3L1.^35^

While direct tumor cell communication via TNTs and TMs is compelling, it is equally important to investigate whether these communication pathways occur between tumor and stromal cells. Astrocytes are the most abundant glia cell type^36^ with several important homeostatic CNS functions,^26^ offering a significant pool for tumor-stroma interactions.^26^ Glial fibrillary acidic protein (GFAP) is the main intermediate filament protein in mature astrocytes^26^ and an established molecular astrocyte marker.^37^ Upon contact to GBM cells, astrocytes are activated showing upregulated GFAP and eventually subvert into tumor-associated astrocytes (TAAs),^26^ which actively contribute to several GBM traits.^25^^,^^26^ TAAs secrete soluble cytokines and growth factors including TGF-β, which heighten immunosuppression and invasion.^25^^,^^26^ They also provide benefits like radioprotection,^26^ adaptation hypoxia^38^ and extracellular matrix (ECM) remodeling.^25^^,^^26^

Several studies reported direct tubular connections between astrocytes and GBM cells.^14^^,^^27^^,^^28^ TNT connections between astrocytes and GBM cells were shown to drive GBM proliferation in a rat model.^28^ TMs between GBM cells and astrocytes have been demonstrated by two independent studies, using either GJ-permeable dyes^27^ or specific labelling with the cytoskeletal marker actin as well as GAP43.^14^ We showed that inhibition of actin polymerization via ­cytochalasin B or knockdown of GAP43 impedes the TM ­network.^14^ Importantly, a functional coupling was indicated both by the presence of synchronized calcium events^27^ and ­mitochondria transfer within the astrocyte-GBM network.^14^ We demonstrated several functional consequences of astrocyte-to-GBM mitochondria transfer, including metabolic reprogramming towards an oxidative phenotype and increased tumorigenicity.^14^

TMs are also part of neuron-to-glioma synapses, a newly discovered synaptic connection between GBM cells and neurons.^39^ GBMs with extensive neuron-to-glioma synapses harbor a neural epigenetic signature and are associated with worse outcomes.^40^ Several neurotransmitters such as glutamate, acetylcholine and dopamine have been implicated in the growth and invasion of GBM through activation of corresponding receptors on TMs.^41^ These observations supported the design of clinical trials for treatment of GBM with repurposed drugs such as the AMPA receptor inhibitor perampanel.^42^

Although the majority of GBM cells form TMs, not all tumor cells are interconnected.^27^ Connected GBM cells represent a stationary tumor cell network, while unconnected cells invade the brain through TMs by adapting neuronal-like cellular mechanisms of invasion with the potential to connect to neurons through synaptic connections.^27^

## Extracellular Vesicles

EVs are secreted as spherical, membrane-clad particles and are separated from cells by their lack of a nucleus.^43^ Unable to independently divide,^43^ the lipid bilayer nonetheless protects its cargo from degradation.^44^ EVs are subdivided into exosomes which are derived from endosomes and micovesicles (MVs) originating from budding of the plasma membrane.^43^ These particles differ in size and estimates diverge in the literature.^43^^,^^45^ Exosomes vary between 30 and 150 nm in diameter, while the corresponding range for MVs has been reported to be from 50 to ∼1300 nm.^46^ Distressed or dying cells do not only release apoptotic bodies (ApoBDs) to the environment,^43^ but also apoptotic extracellular vesicles (apoEVs).^47^ These have been shown to mediate important functional effects on the GBM microenvironment.^47^ From these EV fractions, MVs and ApoBDs contain mitochondria and can be considered as possible mitochondria transfer routes, while exosomes are too small to carry mitochondria.^48^

## Mitochondria and OXPHOS in GBM

Mitochondria are the primary source of OXPHOS which involves the generation and maintenance of a proton gradient as well as the mitochondrial membrane potential (ψΔ_m_). Together, they serve as an intermediate and transient storage of energy prior to ATP production.^49^ As a consequence, mitochondrial transfer results in increased OXPHOS in the recipient population.

Notably, the invasive GBM cell compartment has been linked to OXPHOS.^12^ Compared to the angiogenic GBM cells often found within areas of scant oxygen and nutrients, invasive GBM cells migrate towards regions with comparably higher oxygen levels.^12^ This microenvironment diversity correlates with the use of two distinct metabolic strategies, where the latter cells exhibit an oxidative phenotype.^12^ Reliance on OXPHOS within invasive compared to angiogenic GBM cells was indicated by an increased mitochondrial complex 1 expression and downregulation of glycolysis via the glycolytic proteins LDH-A and GLUT1.^12^ The shift from an invasive to an angiogenic GBM phenotype was also linked to a transition from a proneural to a mesenchymal GBM subtype.^12^

Similar results were reported when comparing treatment-resistant and invasive slow-cycling cells (SCCs) with rapidly proliferating fast-cycling cells (FCCs).^50^ SCCs were identified to rely on mitochondrial OXPHOS as well as lipid metabolism, whereas FCCs depended on glucose metabolism.^50^ Importantly, this dichotomy not only extended to metabolism, but also several phenotypic traits as SCCs were linked to treatment resistance and stem cell features.^50^

Metabolic plasticity has also been noted within glioma stem cells (GSCs) themselves, as a mitochondrial-type GSC was shown to consume more oxygen and uphold a higher ATP content.^9^ These cells were also able to express a glycolytic phenotype with increased lactate production after being exposed to either OXPHOS inhibitors or hypoxia.^9^ This shift was reversible, indicating that a continuous metabolic adaptability may exist in GBM to tackle metabolic stress and different microenvironments.^9^ Others have noted that neither inhibiting glycolysis nor OXPHOS have any significant effect on GSC energy output.^51^ Furthermore, these cells were found to have a higher mitochondrial reserve capacity which corresponded to a higher degree of radioresistance.^51^

More recently, a mitochondrial (MTC) IDH-wildtype GBM subtype has been reported and further underlines an OXPHOS dependence in a GBM subset.^13^ Computational transcriptomic analysis of bulk tumours and single cells focused on shared pathways with clinical significance and subsequently identified MTC GBM as one of four GBM subtypes.^13^ Depending on OXPHOS and exhibiting sensitivity to OXPHOS inhibition, this subtype carried a better prognosis than other GBM subtypes. Importantly, a subset of MTC GBM was found within each of the previously documented GBM subtypes, indicating that OXPHOS dependency is not reflected in the genetic signature and can be present within a subset of GBM cells regardless of their molecular classification.^13^mtDNA is vital for mitochondrial function and encodes 13 essential subunits within the ECT as well as the necessary mechanisms for their translation.^21^^,^^52^^,^^53^ It is also more prone to damage compared to nuclear DNA.^53^ Indeed, mtDNA mutations are associated with mitochondrial dysfunction, while mtDNA integrity is a marker of mitochondrial prowess.^11^ Several observations indicate that mtDNA integrity and thereby mitochondrial function may be important for GBM development.^54–56^ mtDNA depleted GBM cells exhibit slower growth and a reduced tumor initiation capacity.^56^ Furthermore, tumors originating from mtDNA depleted GBM cells reaquired the same mtDNA copy number as controls during tumor development.^56^ There are also relatively few pathogenic mutations in GBM mtDNA ^54^ compared to the high degree of *de novo* mtDNA mutations detected in GBM, and these do not seem to impact the respiratory chain function.^54^

## Mitochondria Transfer

### Physiologic Roles and Mechanisms of HMT

The cell-to-cell transfer of mitochondria is thought to have evolved as a compensatory mechanism to circumvent the effects of age- or disease-associated mutations by importing healthy mitochondria from neighboring cells.^20^ Apart from supplementing recipient cell survival and respiration,^16–18^^,^^57^ the import of mitochondria can dampen innate immune responses in recipient cells.^58^ It may also serve as a mechanism for donor cells to facilitate mitochondrial quality control by discarding dysfunctional mitochondria that are degraded in recipient cells.^59^

**Figure 1.**
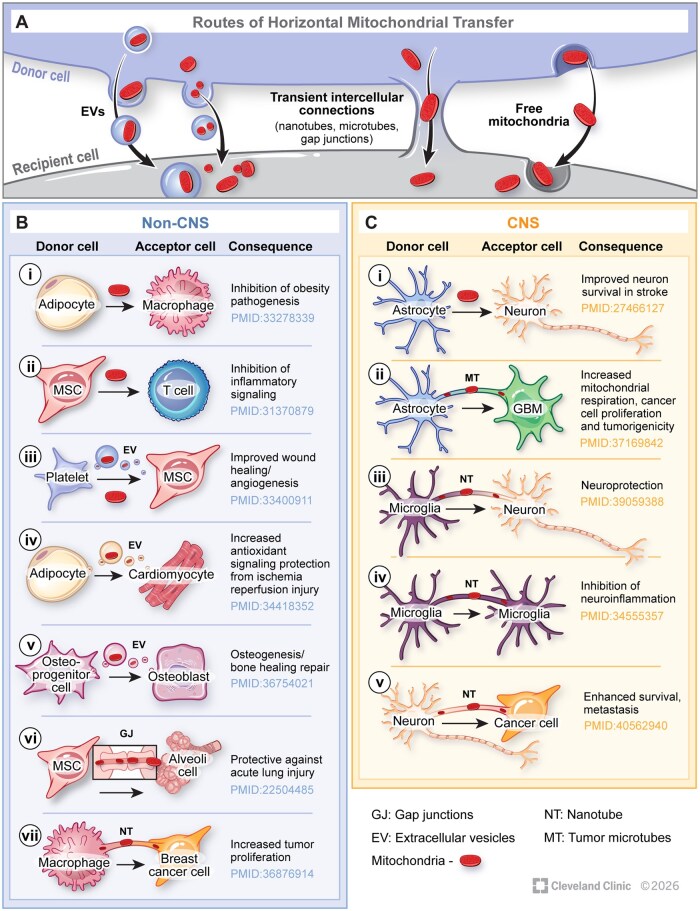
Routes and consequences of HMT. Three major documented routes of HMT have been documented (A); via the sequestration of mitochondria in extracellular vesicles (EVs), through transient intercellular connections such as Tunneling nanotubes (NTs) gap junctions (GJ), or as free mitochondria. Physiologically, horizontal mitochondrial transfer has largely been shown to supplement respiration and/or dampen inflammation in recipient cells. HMT has been documented in non-CNS cell types (B i-vii), as well as CNS cell types (C i-v), culminating in a wide range of physiological consequences.

There are three major routes that cells utilize to effectuate HMT. These include (1) the sequestration of mitochondria/mitochondrial components into extracellular vesicles, which can be secreted by donor cells and transferred via endocytosis into recipient cells, (2) the formation of transient cell-to-cell connections such as nanotubes that ferry mitochondria from donor to recipient cells, as well as (3) the transfer of naked mitochondria, which is a relatively undercharacterized mechanism of mitochondrial transfer (Figure 1A).

### HMT in the CNS and Periphery

Over recent years, multiple lines of investigation in a wide variety of tissues and disease settings have documented mechanisms and consequences of intercellular mitochondrial transfer. These include research performed in non-CNS systems (Figure 1B)^59–65^ as well as CNS niches (Figures 1C).^14^^,^^17^^,^^58^^,^^66^^,^^67^ Overall, three major mechanisms have been described to effectuate HMT; these are transient cell-to-cell connections including TNTs and TMs, the secretion and uptake of EVs, or the secretion and uptake of isolated mitochondria.

### HMT in GBM

Mitochondria transfer in GBM was implied at the onset of TM discovery by noting the ability of mitochondria to travel through these structures, yet not investigated further.^32^ The same authors later indicated that TMs could facilitate directed mitochondria travel, but did not provide further evidence on this matter.^68^ Since then, the association between HMT and direct cellular connections has been rigorously strengthened.^14^^,^^31^^,^^69–73^

**Figure 2.**
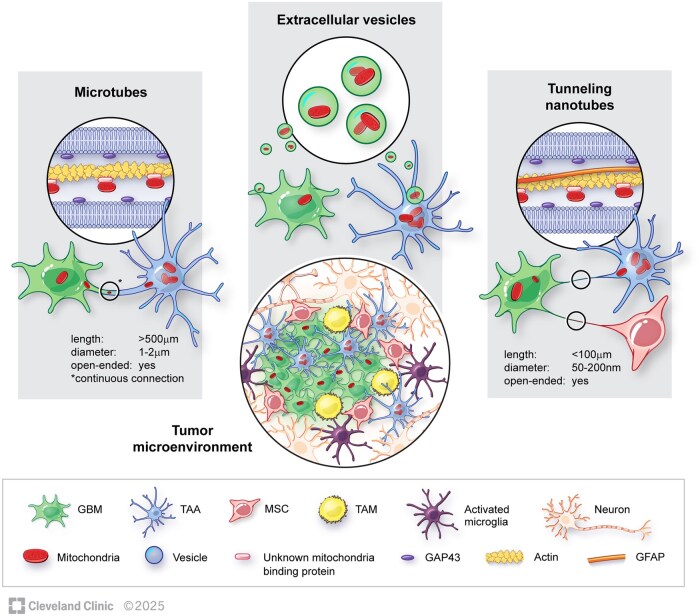
Mechanisms of mitochondria transfer in GBM. Astrocytes are the major mitochondria donors. Major transfer routes are direct cellular connections^14^^,^^31^^,^^69–73^ such as TMs and TNTs. There is less evidence of transfer via EVs in the literature.^69^^,^^74^ GAP43 has been implicated in mitochondria transfer from astrocytes to GBM cells via TMs.^14^ The transfer of mitochondria is an actin dependent process ^14^ with an as of yet unidentified protein mediating the transport of mitochondria.

Research on HMT between GBM and the TME has shown that GBM cells act as mitochondria acceptors.^14^^,^^69–71^^,^^74^ Only one study investigated the role of GBM cells as mitochondria donors where astrocytes served as mitochondria acceptors.^72^ In addition, HMT has also been demonstrated intratumorally between GBM cells.^31^^,^^73^ Most studies focused on mitochondria transfer involving GBM and astrocytes,^14^^,^^70^^,^^72^^,^^74^ yet other cell types have also been discovered as mitochondria donors such as mesenchymal stem cells (MSCs)^71^ and so-called tumor-activated stromal cells (TASCs)^69^ (Figure 2). Furthermore, isolated mitochondria from MSCs have been exogenically transplanted into GSCs, albeit without direct contact between the two cell types.^75^ In contrast to whole organelle transfer, transfer of isolated mtDNA is also possible by incorporation into exosomes. However, only one study addressed this type of transfer showing that mtDNA can be released and exchanged between GBM cells and astrocytes via exosomes.^76^ Thus, in this review, we will focus on whole organelle transfer of mitochondria.

Overall, the literature on TME mitochondria transfer in GBM is scarce and diverges regarding the reported consequences of mitochondrial transmission.^14^^,^^69–72^^,^^74^ Tumor promoting effects like chemoresistance, proliferation, metabolic reprogramming, increased tumorigenicity and malignant adaption of the TME have been described,^14^^,^^69–72^ yet also repressive outcomes such as increased radiosensitivity.^74^ In relation to intratumoral mitochondria transfer, one study reported ambiguous effects of radiation on mitochondria transfer when comparing two invasive areas of the same tumor.^31^ Another study found heightened radioresistance expressed as increased viability after radiation as well as maintenance of oxidative metabolism in irradiated cells.^73^

We explored TME to GBM mitochondrial transfer *in vivo* and found evidence of mitochondria transfer from astrocytes to GBM cells both in a murine GBM model as well as in a GSC xenograft model.^14^ Here, GAP43 and TMs were identified as the underlying mechanism behind contact-dependent mitochondria transfer from astrocytes to GBM cells.^14^ In most studies, the mechanisms governing contact-dependent mitochondria transfer in GBM have not been directly addressed, neither when studying transfer between the TME and GBM^69–72^ or intratumorally.^31^ Yet in other contexts, the transport/transfer of mitochondria through TNTs was shown to involve the calcium-sensitive adaptor protein Miro1,^57^^,^^77^ KIF5 motor proteins and the actin motor protein Myo19.^78^ KIF5 drives anterograde movement on microtubules and Myo19 enables actin-based transport. These motors connect to mitochondria via Miro1/2, calcium-sensing GTPases on the outer mitochondrial membrane that act as regulatory hubs.^78^ This calcium-dependent regulation is particularly important at mitochondria-associated membranes (MAMs), where specialized tethering complexes bring the endoplasmatic reticulum (ER) and mitochondria within 10-30 nm. While it is possible that these proteins play a role in GBM mitochondria transfer, this has yet to be experimentally confirmed. Using a soluble transfer route of mitochondria to GBM, that had been isolated from astrocytes, one study implicates NAD-CD38-cADPR-Ca^2+^ signalling in relation to HMT,^74^ which has been described previously to be the ­mechanism of transfer between astrocytes and neurons after stroke.^17^

### Mechanisms of HMT

In general, most of the literature on GBM mitochondria transfer builds on the discovery of GBM network formation published in 2015^32^ by highlighting direct intercellular connections between mitochondrial donors and acceptors.^14^^,^^31^^,^^69–73^ Indeed, some studies have found evidence of more than one connection subtype occuring simultaneously.^31^ Importantly, intercellular connections have been documented not only intertumorally,^31^^,^^73^ but also between GBM cells and astrocytes,^14^^,^^70^ thereby indicating heightened network complexity and a direct pathway between malignant cells and the TME.^67^

Apart from physical connections, several studies emphasized the importance of indirect or soluble routes in cellular CNS communication.^17^^,^^79^ Within the field of GBM mitochondrial transfer, the results on this transfer type are more inconclusive.^14^^,^^69^^,^^74^^,^^76^ We did not find a significant mitochondria transfer when physically separating astrocyte donors and GBM acceptors,^14^ although others have found evidence of transfer via EVs^69^ and endocytosis^74^; these differences could be attributed to variations in the cell culture systems employed or the donor cell type.

Notably, several simultaneous mitochondrial transfer routes have been described including direct and indirect contact between cells as well as cannibalism.^69^ The latter was observed via electron and light microscopy, showing TASCs being engulfed by GBM cells.^69^ This may indicate that mitochondria transfer is possible through several pathways simultaneously, prompting the speculation that the precise mode of transfer may determine the downstream cellular effect.^80^ Alternatively, the exact HMT path may be specific to the cellular context and be determined by the cells involved, as HMT between astrocytes and GBM cells has been deemed to be contact-dependent^14^^,^^70^^,^^72^ while HMT from astrocytes to neurons is mediated by a soluble route.^17^

The first appearance of mitochondrial transfer in GBM cells was in a protocol published in 2017,^75^ directly building on a 2015 paper using identical principles on acceptor breast cancer cells (MDA-MB-231).^81^ In these two protocols, fluorescently labelled mitochondria from MSCs were isolated and added to green CellTracker-^81^ or green vital dye-labelled acceptor cells.^75^ This technique was termed MitoCeption.^75^^,^^81^ Using this technique, three methods for validating the transfer from MSCs to GSCs were described, including confocal imaging, fluorescence-activated cell sorting (FACS) and the estimation of MSC mtDNA in GSC acceptor cells.^75^ Importantly, these methods are only meant to verify a successful transfer, paving the way for functional analysis of acceptor GSCs.^75^ This was exemplified by transfer of isolated astrocyte mitochondria to acceptor GSCs, demonstrating a skewing towards the proliferative G2/M phase by cell cycle analysis.^14^

*In vivo* mitochondrial transfer was shown by transplantation of isolated mitochondria from astrocytes to U87 ­xenografts.^74^ This led to inhibited proliferation and radiosensitization in nude mice.^74^ In parallel, *in vitro* mitochondria transplantation was performed on starved U87 monocultures, where NAD-CD38-cADPR-Ca^2+^ signaling was unravelled as the mechanism for mitochondria internalization.^74^ Following transplantation, increased aerobic respiration, reactivation of the apoptotic pathway, radiosensitization and inhibited proliferation were reported as downstream consequences.^74^

In 2019, the first confirmation of HMT from astrocytes to GBM cells was based on co-cultures where non-neoplastic mitochondria travelled via TNTs.^70^ These membranous structures spanned long distances (up to 200µm), containing F-actin and were formed by the astrocytes^70^ (Figure 2). Mitochondria transfer was also observed in a 3D *in vitro* model, postulated to better simulate human *in vivo* conditions.^70^ TNTs are the most frequently described mechanism for mitochondria transfer in gliomas and are formed between glioma cells and various cells in the TME such as astrocytes, mesenchymal stem cells and othe tumor associated stromal cells.^31^^,^^69–72^

Besides TNTs, TMs are the major pathway for HMT in gliomas owing to the fact that these structures are unique for this tumor type^32^ (Figure 2). Using two distinct *in vivo* models, we demonstrated that the intermediate filament protein GAP43 is an important element for mitochondria transfer between astrocytes and GBM cells via TMs.^14^ The murine model was based on orthotopically implanting green fluorescent protein (GFP)-expressing mouse GBM cells into transgenic mice where host cell mitochondria were labelled with mKate2.^14^ In the xenograft model, normal rat brain cells were transduced by lentiviral vectors carrying a mitochondrial tag followed by injection of human-derived GFP-labelled GSCs.^14^ In both models, we confirmed host mitochondria within acceptor GBM cells as well as mitochondria in transit along intercellular connections by confocal microscopy.^14^ Brain-resident cells were the major donors of mitochondria to GBM cells *in vivo*, and of these, astrocytes were identified to have the highest mitochondria transfer rate based on *in vitro* co-culture experiments.^14^ The transfer was dependent on physical contact, and the membrane protrusions were identified as TMs due to the presence of GAP43.^14^

In another study, mitochondria transfer was explored within a cellular network model referred to as *de novo*-induced cellular network (iNET), most likely dependent on TMs.^73^ Here, mitochondria trafficking was observed in iNET projections between MitoTracker-labelled donor cells and GFP-labelled acceptor cells using confocal time lapse ­imaging.^73^ Importantly, a concurrent oxidative metabolic reprogramming and radioresistance was indicated, confirming functional consequences of contact-dependent mitochondria transfer between tumor cells.^73^

Electron microscopic observation of EVs containing mitochondria indicate vesicles as a possible transfer route^69^ (Figure 2), yet the subsequent endocytosis or direct fusion necessary for the completion of this process^82^ was not investigated further. A soluble transfer route has also been documented for mtDNA in both astrocytes and GBM, albeit final confirmation of exchange was not reported.^76^ The fraction of mtDNA carried by exosomes was low (about 5%) compared to the total amount of secreted mtDNA,^76^ suggesting only a minor contact-independent route for mtDNA in GBM via EVs. Thus, mechanistic insights into a possible EV-mediated transfer of whole mitochondria and/or mtDNA in GBM remain elusive.

### Functional Impact of HMT

Although the physiological consequence and underlying mechanisms of HMT are debated,^20^^,^^21^ early *in vivo* experiments indicated functional restitution of injured acceptor cells.^57^^,^^64^ Supporting these observations is the fact that ­cellular stress stimulates HMT.^20^^,^^21^ More broadly, HMT may be important in tissue homeostasis,^83^ as cell differentiation,^84^ intercellular signalling^85^ and immune function^86^ are all affected by mitochondria transfer. Mitochondria quality control might also be influenced by HMT,^87^ as cells receive and discard mitochondria to maintain a pristine organelle pool.^87^^,^^88^ Notably, although MSCs or other stem cells are frequently used as mitochondrial donors,^16^^,^^69^^,^^77^^,^^86^^,^^89–91^ HMT’s importance goes beyond stem cell therapy.^17^^,^^79^^,^^90^^,^^92^

### Metabolism

Recent evidence indicate less reliance on aerobic glycolysis in GBM than previously assumed,^10^ and there is a deviant metabolism of carbohydrates as well as other biochemical compounds like lipids, nucleotides and glutamine.^9^^,^^10^ Consequently, aerobic glycolysis,^10^ OXPHOS^9^^,^^10^^,^^12^ and fatty acid oxidation^10^ are utilized by different GBM subpopulations. Indeed, OXPHOS is now viewed as important in GBM, at least within a cellular subset.^12^^,^^13^^,^^24^

**Figure 3.**
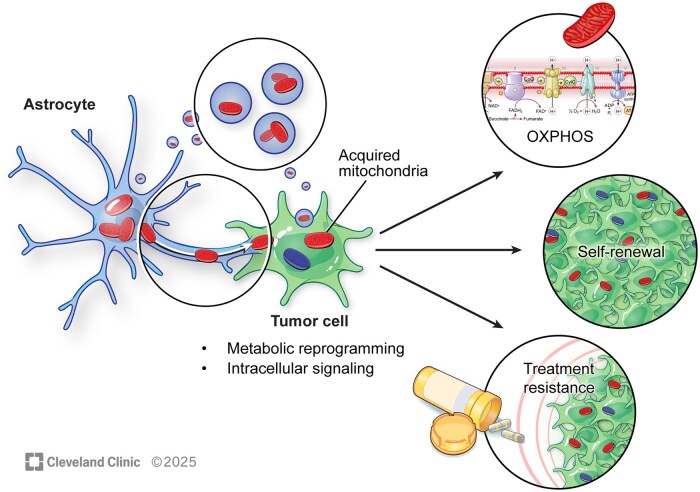
Functional impact of mitochondria transfer. Mitochondria transfer from astrocytes promotes proliferation, self-renewal, metabolic reprogramming and treatment resistance.

Seahorse assays are used to measure oxidative metabolism in GBM cells after accepting mitochondria,^14^^,^^71–73^ as well as the glycolytic rate.^69^ Notably, several metabolic parameters may be simultaneously affected by HMT, as GBM cells exhibited an increased glycolytic signature and only partial OXPHOS upregulation following co-culture with TME cells.^69^ This may depend on the donor cell type as we observed that GBM cells exhibit metabolic reprogramming towards an oxidative phenotype after mitochondria donation from astrocytes *in vitro*^14^ (Figure 3). Similar *in vitro* results were obtained using MSCs as mitochondria donors.^71^ In both reports, a dose-dependent and stepwise increase in OXPHOS followed the degree of mitochondrial uptake.^14^^,^^71^ Only one study investigated the metabolic impact of mitochondria transfer from GBM cells to astrocytes. Here, astrocytes receiving mitochondria from GBM cells adapted to a tumor-like metabolism and showed increased survival under hypoxic conditions.^72^

Exploring intratumoral HMT, interconnected malignant cells relied on a more oxidative metabolism following a high dose of radiation (20 Gy) when compared to unconnected controls.^73^ Importantly, increased ATP output has been reported in acceptor GBM cells following mitochondria ­transfer.^14^^,^^71^ The ATP synthase subunit ATP5A was also upregulated in acceptor GSCs, supporting the hypothesis that mitochondria transfer induces an oxidative phenotype.^14^

Notably, OXPHOS has been detected in therapy resistant GSCs,^9^ while essential amino acids, α-ketoglutarate, glutamate and glutathione are all upregulated after mitochondria transfer.^14^ The profile of upregulated metabolites indicates a higher *de novo* nucleotide synthesis and an increased proliferation rate as well as being suggestive of greater therapy resistance.^93^^,^^94^  The role of glutamine in tumour metabolism was also highlighted in a comparison between different energy sources in GBM cells and astrocytes.^72^ Here, glutamine was utilized exclusively by tumours, while both tumour cells and astrocytes relied on glucose and fatty acids.^72^ Interestingly, astrocytes were shown to consume glutamine after only 12-24 hours in co-culture with GBM cells, thereby shifting towards a tumour-like metabolism with resistance against hypoxia.^72^ This metabolic shift was associated with mitochondria transfer from tumour cells to astrocytes, as the inhibition of this transfer also prevented glutamine dependency.^72^ Such a shift from glucose-dependency towards glutamine-dependency has also been reported by others.^71^ Using isotope-profiling experiments after mitochondria transfer, heightened glutamine consumption at the expense of glucose utilisation indicated a nutrient shift within acceptor GBM cells.^71^ The breakdown of glutamine was also skewed towards reductive carboxylation rather than glutaminolysis, presenting a possible therapeutic target.^71^

OXPHOS, and hence HMT, is inherently linked to redox balance and, under stress conditions or organellar dysfunction, it can lead to increased levels of reactive oxygen species (ROS). Importantly, ROS may serve as signaling molecules,^11^^,^^95^ suggesting that HMT can alter not only cellular respiration and metabolism but also intracellular signaling. In breast cancer models it was shown that macrophages selectively transfer dysfunctional mitochondria which stimulate a proliferative response in recipient cancer cells through ERK signaling.^95^ In the context of GBM, ERK has been shown to play a central role in driving GSC formation by promoting pluripotency marker expression and supporting self-renewal.^96^ While it remains to be seen how HMT driven redox imbalance affects GBM cellular states, modulation of intracellular signaling emerges as a potential pathway.

### Tumor Growth

HMT has been demonstrated to affect GBM proliferation.^14^^,^^69^ We observed that a higher fraction of cells was skewed towards the G2/M phase of the cell cycle following mitochondria transfer from astrocytes *in vitro* as well as within two different cell lines in a mouse model *in vivo*^14^ (Figure 3). Importantly, heat-killed mitochondria did not show cellular uptake at any discernible rate which strongly refutes the ­possibility of passive uptake of mitochondria debris.^14^ Furthermore, isolated and viable mitochondria induced a G2/M shift in GBM cells, indicating that astrocytes *per se* did not contribute decisively to these proliferative effects.^14^ Interestingly, the acquisition of mitochondria from astrocytes produced an elevated degree of stemness in GBM cells,^14^ measured as a higher stem cell frequency *in vitro* and *in vivo* (Figure 3). This was further supported by the upregulation of the self-renewal transcription factor OCT4 as well as an increased tumorigenicity *in vivo.*^14^ Another study investigated how the donation of stromal mitochondria contributed to GBM proliferation.^69^ Using TASCs as mitochondria donors and mtDNA depleted (p^0^) GBM cells as acceptors, increased GBM proliferation was detected 7 days after coculture when compared to controls.^69^ After coculturing TASCs with non-depleted GBM cells, there was an increase in proliferation when compared to non-depleted GBM monocultures, indicating that the proliferative effect from the microenvironment was not dependent on previous mitochondria depletion.^69^

### Treatment Resistance

Since HMT affects proliferation of gliomas and promotes metabolic plasticity, it was an obvious suggestion that transfer may also affect therapy resistance. After co-culturing astrocytes with GBM cells, chemoresistance against temozolomide was detected alongside a reactive astrocyte ­phenotype with GFAP upregulation.^70^ The effect was contact-dependent, and astrocyte mitochondria were concurrently transferred via TNTs to GBM cells^70^ (Figure 3). Chemoresistance has also been shown following mitochondria transfer from MSCs to GBM cells via TNTs.^71^ Mitochondria from donor MSCs were detected within GBM acceptor cells as well as interconnecting TNTs, while also detecting organelle movement inside the latter structures.^71^

Downstream metabolic reprogramming was identified as an underlying temozolomide (TMZ) resistance mechanism, more specifically related to a higher turnover of the metabolite orotate in GBM cells with donor mitochondria.^71^ Inhibition of orotate production by Brequinar (BRQ) also reinstated TMZ sensitivity in these GBM cells.^71^ Few reports have specifically investigated the effects of radiotherapy, and the described effects differ.^73^^,^^74^ In one study, mitochondria transplantation was determined to be a possible radiosensitizer *in vitro* and *in vivo.*^74^ In another study, HMT within a tumor network was related to radioresistance, although no causal link was established.^73^ There was also no clear-cut effect when focusing on the effect of irradiation on mitochondria transfer, rather than the effect of mitochondria transfer in relation to radioresistance.^31^

### Immune Evasion

There is increasing evidence that HMT in GBM is not exclusively monodirectional but can also occur from GBM cells back to astrocytes.^72^ This raises the possibility that astrocytes may subsequently pass these mitochondria on to other cells, or that additional cellular components of the tumor microenvironment (TME) are directly targeted by HMT.

Such processes could contribute to immune evasion mechanisms similar to those described in non–small cell lung cancer (NSCLC) and melanoma.^65^ Mitochondria harboring mtDNA mutations were taken up by tumor-infiltrating lymphocytes, leading to mitochondrial dysfunction and impaired antitumor immunity in vivo.^65^ A comparable mechanism may also operate in GBM, particularly given that tumor cells can acquire mitochondria from microglial cells.^14^ This would require further research into how this mitochondria transfer is mediated and if it allows for bidirectional transfer. In such a scenario, GBM cells could impair microglial function by supplying dysfunctional mitochondria in exchange for functional ones, thereby reducing local antitumor capacity. Nevertheless, any conclusions regarding the biological relevance and functional consequences of HMT on immune evasion in GBM remain highly speculative at this stage.

### Translational Implications of HMT

The importance of HMT in GBM progression is supported by preclinical studies showing it to convey a metabolic shift towards glutamine dependency, elevated nucleotide synthesis^71^ and respiration.^14^ Furthermore, it is associated with increased TMZ resistance^71^ as well as a more tumorigenic and proliferative phenotype.^14^

The reestablishment of OXPHOS in GBM has also been linked to pro-malignant traits.^92^ In this study, OXPHOS stimulated activation of the enzyme dihydroorotate dehydrogenase (DHODH) which enabled release from cell-cycle arrest through its essential role in pyrimidine synthesis.^92^ This enzyme has recently been identified as a promising target.^19^^,^^92^^,^^97^^,^^98^ Reactivation of DHODH and thereby *de novo* pyrimidine synthesis restored GBM respiration via mitochondria transfer from TME astrocytes.^19^ The essential role of OXPHOS restoration in tumorigenesis also extends to other cancer types, where DHODH and *de novo* pyrimidine synthesis reverse cell-cycle arrest.^92^

Targeting OXPHOS in GBM is another therapeutic strategy,^24^^,^^99^ and may particularly affect GSCs and/or other GBM cell populations with increased OXPHOS reliance and/or OXPHOS defects. The complex V (ATP synthase) inhibitor Gboxin specifically blocks OXPHOS.^24^ Treatment effects using this inhibitor were observed in GBM allografts and patient-derived xenografts, indicating O_2_-dependence in at least a GBM subset.^24^^,^^99^

HMT is mediated by TMs,^14^ while TM formation is dependent on the structural protein GAP43.^32^ GAP43 knockdown led to structurally impaired TMs, reduced tumour dissemination, fewer TM connections, decreased tumour size, increased radiosensitivity and improved survival *in vivo.*^32^ However, there are to our knowledge no current efforts targeting this protein. This may be due to a high risk of side-effects caused by its broad expression and close association with neuronal development.^32^ The TGF-β/TSP1 signaling axis is another mediator of TM formation,^33^ however, the role of this pathway in mitochondria transfer is unexplored. The current knowledge on mechanisms mediating HMT via TMs is generally scarce and future efforts are needed to unravel more specific mechanisms that could be translated into targeted treatments.

### Future Perspectives

Mitochondria transfer in GBM has gained increased attention in recent years, both as an intratumoral phenomenon as well as a process occurring in concert with the TME. Subsequent research has confirmed mitochondria transfer to be a widespread activity, taking place both *in vivo*, *ex vivo* and *in vitro*. So far, the emerging evidence mainly points to a contact-dependent transfer mechanism, either through TNTs or TMs, leading to pro-tumorigenic downstream effects in acceptor GBM cells.

However, several unanswered questions remain. Little is currently known regarding donor cell characteristics, the governing genes behind HMT and the effector signals controlling mitochondria transfer. Also, to further characterize the downstream effects in acceptor cells will be paramount, particularly since mitochondria themselves are involved in such a wide variety of cellular functions. The influence of HMT on redox balance across different tumor microenvironments—and its potential intersection with radiotherapy response and treatment timing—is another unresolved area and warrants further investigation. While bioinformatic approaches have been proposed to infer mitochondria transfer in patient specimens,^100^ it remains unclear whether these pipelines can be applied in the bidirectional transfer setting of GBM. Thus, the frequency and downstream effects of mitochondria transfer in patients remains unknown, and must be inferred from model systems until these challenges are overcome. Currently, this also limits knowledge about potential patient stratification for future therapies targeting HMT and whether only specific subsets of patients will respond. Further, it remains to be shown if HMT is more effective in specific GBM cell subtypes such as GSCs or more differentiaited tumor cells. It also remains unclear how the transfer of dysfunctional or damaged mitochondria from GBM cells to stromal cells affects the TME. Utilization of genetically encoded mitochondria labeling,^14^ lineage tracing,^67^ and orthogonal assays will be critical to strengthening conclusions. Further research is urgently needed to remedy our current lack of knowledge, and may also provide diagnostic or therapeutic gains in the future.

## References

[1] Molinaro AM , Taylor JW, Wiencke JK, Wrensch MR. Genetic and ­molecular epidemiology of adult diffuse glioma. Nat Rev Neurol. 2019;15:405-417. 10.1038/s41582-019-0220-2 31227792 PMC7286557

[2] Alexander BM , Cloughesy TF. Adult glioblastoma. J Clin Oncol. 2017;35:2402-2409. 10.1200/JCO.2017.73.0119 28640706

[3] Osuka S , Van Meir EG. Overcoming therapeutic resistance in glioblastoma: the way forward. J Clin Invest. 2017;127:415-426. 10.1172/JCI89587 28145904 PMC5272196

[4] Friedmann-Morvinski D. Glioblastoma heterogeneity and cancer cell plasticity. Crit Rev Oncog. 2014;19:327-336. 10.1615/critrevoncog.2014011777 25404148

[5] Chabon JJ , Simmons AD, Lovejoy AF, et al Circulating tumour DNA profiling reveals heterogeneity of EGFR inhibitor resistance mechanisms in lung cancer patients. Nat Commun. 2016;7:11815. 10.1038/ncomms11815 27283993 PMC4906406

[6] Cuddapah VA , Robel S, Watkins S, Sontheimer H. A neurocentric perspective on glioma invasion. Nat Rev Neurosci. 2014;15:455-465. 10.1038/nrn3765 24946761 PMC5304245

[7] de Gooijer MC , Guillen Navarro M, Bernards R, Wurdinger T, van Tellingen O. An experimenter’s guide to glioblastoma invasion pathways. Trends Mol Med. 2018;24:763-780. 10.1016/j.molmed.2018.07.003 30072121

[8] Garcia JH , Jain S, Aghi MK. Metabolic drivers of invasion in glioblastoma. Front Cell Dev Biol. 2021;9:683276. 10.3389/fcell.2021.683276 34277624 PMC8281286

[9] Shibao S , Minami N, Koike N, et al Metabolic heterogeneity and plasticity of glioma stem cells in a mouse glioblastoma model. Neuro Oncol. 2018;20:343-354. 10.1093/neuonc/nox170 29016888 PMC5817950

[10] Caniglia JL , Jalasutram A, Asuthkar S, et al Beyond glucose: alternative sources of energy in glioblastoma. Theranostics. 2021;11:2048-2057. 10.7150/thno.53506 33500708 PMC7797684

[11] Strickland M , Stoll EA. Metabolic reprogramming in glioma. Front Cell Dev Biol. 2017;5:43. 10.3389/fcell.2017.00043 28491867 PMC5405080

[12] Talasila KM , Rosland GV, Hagland HR, et al The angiogenic switch leads to a metabolic shift in human glioblastoma. Neuro Oncol. 2017;19:383-393. 27591677 10.1093/neuonc/now175 PMC5464376

[13] Garofano L , Migliozzi S, Oh YT, et al Pathway-based classification of glioblastoma uncovers a mitochondrial subtype with therapeutic vulnerabilities. Nat Cancer. 2021;2:141-156. 10.1038/s43018-020-00159-4 33681822 PMC7935068

[14] Watson DC , Bayik D, Storevik S, et al GAP43-dependent mitochondria transfer from astrocytes enhances glioblastoma tumorigenicity. Nat Cancer. 2023;4:648-664. 10.1038/s43018-023-00556-5 37169842 PMC10212766

[15] Dong LF , Rohlena J, Zobalova R, et al Mitochondria on the move: ­horizontal mitochondrial transfer in disease and health. J Cell Biol. 2023;222. 10.1083/jcb.202211044 PMC9960264 36795453

[16] Spees JL , Olson SD, Whitney MJ, Prockop DJ. Mitochondrial transfer between cells can rescue aerobic respiration. Proc Natl Acad Sci USA. 2006;103:1283-1288. 10.1073/pnas.0510511103 16432190 PMC1345715

[17] Hayakawa K , Esposito E, Wang X, et al Transfer of mitochondria from astrocytes to neurons after stroke. Nature. 2016;535:551-555. 10.1038/nature18928 27466127 PMC4968589

[18] Tan AS , Baty JW, Dong LF, et al Mitochondrial genome acquisition restores respiratory function and tumorigenic potential of cancer cells without mitochondrial DNA. Cell Metab. 2015;21:81-94. 10.1016/j.cmet.2014.12.003 25565207

[19] Brisudova P , Stojanovic D, Novak J, et al Functional mitochondrial ­respiration is essential for glioblastoma tumour growth. Oncogene. 2025;44:2588-2603. 10.1038/s41388-025-03429-6 40325182 PMC12277175

[20] Valenti D , Vacca RA, Moro L, Atlante A. Mitochondria can cross cell boundaries: an overview of the biological relevance, pathophysiological implications and therapeutic perspectives of intercellular mitochondrial transfer. Int J Mol Sci. 2021;22:8312. 10.3390/ijms22158312 34361078 PMC8347886

[21] Torralba D , Baixauli F, Sanchez-Madrid F. Mitochondria know no boundaries: mechanisms and functions of intercellular mitochondrial transfer. Front Cell Dev Biol. 2016;4:107. 10.3389/fcell.2016.00107 27734015 PMC5039171

[22] Guntuku L , Naidu VG, Yerra VG. Mitochondrial dysfunction in gliomas: pharmacotherapeutic potential of natural compounds. Curr Neuro­pharmacol. 2016;14:567-583. 10.2174/1570159x14666160121115641 PMC4981742 26791479

[23] Arismendi-Morillo GJ , Castellano-Ramirez AV. Ultrastructural mitochondrial pathology in human astrocytic tumors: potentials implications pro-therapeutics strategies. J Electron Microsc (Tokyo). 2008;57:33-39. 10.1093/jmicro/dfm038 18230641

[24] Shi Y , Lim SK, Liang Q, et al Gboxin is an oxidative phosphorylation inhibitor that targets glioblastoma. Nature. 2019;567:341-346. 10.1038/s41586-019-0993-x 30842654 PMC6655586

[25] Nieland L , Morsett LM, Broekman MLD, Breakefield XO, Abels ER. Extracellular vesicle-mediated bilateral communication between glioblastoma and astrocytes. Trends Neurosci. 2021;44:215-226. 10.1016/j.tins.2020.10.014 33234347 PMC7904598

[26] Brandao M , Simon T, Critchley G, Giamas G. Astrocytes, the rising stars of the glioblastoma microenvironment. Glia. 2019;67:779-790. 10.1002/glia.23520 30240060

[27] Venkataramani V , Yang Y, Schubert MC, et al Glioblastoma hijacks neuronal mechanisms for brain invasion. Cell. 2022;185:2899-2917.e31. 10.1016/j.cell.2022.06.054. 35914528

[28] Zhang L , Zhang Y. Tunneling nanotubes between rat primary astrocytes and C6 glioma cells alter proliferation potential of glioma cells. Neurosci Bull. 2015;31:371-378. 10.1007/s12264-014-1522-4 25913038 PMC5563692

[29] Rustom A , Saffrich R, Markovic I, Walther P, Gerdes HH. Nanotubular highways for intercellular organelle transport. Science. 2004;303:1007-1010. 14963329 10.1126/science.1093133

[30] Zamberlan M , Semenzato M. Mechanisms of mitochondrial transfer through TNTs: from organelle dynamics to cellular crosstalk. Int J Mol Sci. 2025;26:10581. 10.3390/ijms262110581 41226616 PMC12607586

[31] Pinto G , Saenz-de-Santa-Maria I, Chastagner P, et al Patient-derived glioblastoma stem cells transfer mitochondria through tunneling nanotubes in tumor organoids. Biochem J. 2021;478:21-39. 10.1042/BCJ20200710 33245115 PMC7800365

[32] Osswald M , Jung E, Sahm F, et al Brain tumour cells interconnect to a functional and resistant network. Nature. 2015;528:93-98. 10.1038/nature16071 26536111

[33] Joseph JV , Magaut CR, Storevik S, et al TGF-beta promotes microtube formation in glioblastoma through thrombospondin 1. Neuro Oncol. 2021; 10.1093/neuonc/noab212 PMC8972291 34543427

[34] Jung E , Osswald M, Blaes J, et al Tweety-Homolog 1 drives brain ­colonization of gliomas. J Neurosci. 2017;37:6837-6850. 10.1523/JNEUROSCI.3532-16.2017 28607172 PMC6705725

[35] Hai L , Hoffmann DC, Wagener RJ, et al A clinically applicable connectivity signature for glioblastoma includes the tumor network driver CHI3L1. Nat Commun. 2024;15:968. 10.1038/s41467-024-45067-8 38320988 PMC10847113

[36] Jakel S , Dimou L. Glial cells and their function in the adult brain: a journey through the history of their ablation. Front Cell Neurosci. 2017;11:24. 10.3389/fncel.2017.00024 28243193 PMC5303749

[37] Sofroniew MV , Vinters HV. Astrocytes: biology and pathology. Acta Neuropathol. 2010;119:7-35. 10.1007/s00401-009-0619-8 20012068 PMC2799634

[38] Pantazopoulou V , Jeannot P, Rosberg R, Berg TJ, Pietras A. Hypoxia-Induced reactivity of Tumor-Associated astrocytes affects glioma cell properties. Cells. 2021;10:613. 10.3390/cells10030613 33802060 PMC7999295

[39] Venkataramani V , Tanev DI, Strahle C, et al Glutamatergic synaptic input to glioma cells drives brain tumour progression. Nature. 2019;573:532-538. 10.1038/s41586-019-1564-x 31534219

[40] Drexler R , Khatri R, Sauvigny T, et al A prognostic neural epigenetic signature in high-grade glioma. Nat Med. 2024;30:1622-1635. 10.1038/s41591-024-02969-w 38760585 PMC11186787

[41] Tetzlaff SK , Reyhan E, Layer N, et al Characterizing and targeting ­glioblastoma neuron-tumor networks with retrograde tracing. Cell. 2025;188:390-411.e36. 10.1016/j.cell.2024.11.002e336. 39644898

[42] Heuer S , Burghaus I, Gose M, et al PerSurge (NOA-30) phase II trial of perampanel treatment around surgery in patients with progressive glioblastoma. BMC Cancer. 2024;24:135. 10.1186/s12885-024-11846-1 38279087 PMC10811925

[43] Gourlay J , Morokoff AP, Luwor RB, Zhu HJ, Kaye AH, Stylli SS. The emergent role of exosomes in glioma. J Clin Neurosci. 2017;35:13-23. 10.1016/j.jocn.2016.09.021 27771233

[44] Maas SLN , Breakefield XO, Weaver AM. Extracellular vesicles: Unique intercellular delivery vehicles. Trends Cell Biol. 2017;27:172-188. 10.1016/j.tcb.2016.11.003 27979573 PMC5318253

[45] Yekula A , Yekula A, Muralidharan K, Kang K, Carter BS, Balaj L. Extracellular vesicles in glioblastoma tumor microenvironment. Front Immunol. 2019;10:3137. 10.3389/fimmu.2019.03137 32038644 PMC6990128

[46] Xu R , Rai A, Chen M, Suwakulsiri W, Greening DW, Simpson RJ. Extracellular vesicles in cancer - implications for future improvements in cancer care. Nat Rev Clin Oncol. 2018;15:617-638. 10.1038/s41571-018-0036-9 29795272

[47] Pavlyukov MS , Yu H, Bastola S, et al Apoptotic cell-derived extracellular vesicles promote malignancy of glioblastoma via intercellular transfer of splicing factors. Cancer Cell. 2018;34:119-135.e10. 10.1016/j.ccell.2018.05.012e110 29937354 PMC6048596

[48] Manickam DS , Pinky PP, Khare P. Extracellular vesicle-mediated mitochondria delivery: Premise and promise. J Cereb Blood Flow Metab. 2026;46:306-321. 10.1177/0271678X251349304 40498574 PMC12158986

[49] Zorova LD , Popkov VA, Plotnikov EY, et al Mitochondrial membrane potential. Anal Biochem. 2018;552:50-59. 10.1016/j.ab.2017.07.009 28711444 PMC5792320

[50] Hoang-Minh LB , Siebzehnrubl FA, Yang C, et al Infiltrative and drug-resistant slow-cycling cells support metabolic heterogeneity in ­glioblastoma. Embo J. 2018;37:e98772. 10.15252/embj.201798772 30322894 PMC6276884

[51] Vlashi E , Lagadec C, Vergnes L, et al Metabolic state of glioma stem cells and nontumorigenic cells. Proc Natl Acad Sci USA. 2011;108:16062-16067. 10.1073/pnas.1106704108 21900605 PMC3179043

[52] Wallace DC. Mitochondria and cancer. Nat Rev Cancer. 2012;12:685-698. 10.1038/nrc3365 23001348 PMC4371788

[53] Annesley SJ , Fisher PR. Mitochondria in health and disease. Cells. 2019;8:680. 10.3390/cells8070680 31284394 PMC6678092

[54] Vidone M , Clima R, Santorsola M, et al A comprehensive characterization of mitochondrial DNA mutations in glioblastoma multiforme. Int J Biochem Cell Biol. 2015;63:46-54. 10.1016/j.biocel.2015.01.027 25668474

[55] Lloyd RE , Keatley K, Littlewood DT, et al Identification and functional prediction of mitochondrial complex III and IV mutations associated with glioblastoma. Neuro Oncol. 2015;17:942-952. 10.1093/neuonc/nov020 25731774 PMC4474231

[56] Dickinson A , Yeung KY, Donoghue J, et al The regulation of mitochondrial DNA copy number in glioblastoma cells. Cell Death Differ. 2013;20:1644-1653. 10.1038/cdd.2013.115 23995230 PMC3824586

[57] Ahmad T , Mukherjee S, Pattnaik B, et al Miro1 regulates intercellular mitochondrial transport & enhances mesenchymal stem cell rescue efficacy. Embo J. 2014;33:994-1010. 10.1002/embj.201386030 24431222 PMC4193933

[58] Scheiblich H , Dansokho C, Mercan D, et al Microglia jointly degrade fibrillar alpha-synuclein cargo by distribution through tunneling nanotubes. Cell. 2021;184:5089-5106.e21. 10.1016/j.cell.2021.09.007e5021 34555357 PMC8527836

[59] Crewe C , Funcke JB, Li S, et al Extracellular vesicle-based interorgan transport of mitochondria from energetically stressed adipocytes. Cell Metab. 2021;33:1853-1868.e11. 10.1016/j.cmet.2021.08.002e1811 34418352 PMC8429176

[60] Brestoff JR , Wilen CB, Moley JR, et al Intercellular mitochondria transfer to macrophages regulates white adipose tissue homeostasis and is impaired in obesity. Cell Metab. 2021;33:270-282.e8. 10.1016/j.cmet.2020.11.008e278. 33278339 PMC7858234

[61] Luz-Crawford P , Hernandez J, Djouad F, et al Mesenchymal stem cell repression of Th17 cells is triggered by mitochondrial transfer. Stem Cell Res Ther. 2019;10:232. 10.1186/s13287-019-1307-9 31370879 PMC6676586

[62] Levoux J , Prola A, Lafuste P, et al Platelets facilitate the Wound-Healing capability of mesenchymal stem cells by mitochondrial transfer and metabolic reprogramming. Cell Metab. 2021;33:283-299.e9. 10.1016/j.cmet.2020.12.006e289. 33400911

[63] Suh J , Kim NK, Shim W, et al Mitochondrial fragmentation and donut formation enhance mitochondrial secretion to promote osteogenesis. Cell Metab. 2023;35:345-360.e7. 10.1016/j.cmet.2023.01.003e347. 36754021

[64] Islam MN , Das SR, Emin MT, et al Mitochondrial transfer from bone-marrow-derived stromal cells to pulmonary alveoli protects against acute lung injury. Nat Med. 2012;18:759-765. 10.1038/nm.2736 22504485 PMC3727429

[65] Ikeda H , Kawase K, Nishi T, et al Immune evasion through mitochondrial transfer in the tumour microenvironment. Nature. 2025;638:225-236. 10.1038/s41586-024-08439-0 39843734 PMC11798832

[66] Scheiblich H , Eikens F, Wischhof L, et al Microglia rescue neurons from aggregate-induced neuronal dysfunction and death through tunneling nanotubes. Neuron. 2024;112:3106-3125.e8. 10.1016/j.neuron.2024.06.029e3108. 39059388

[67] Hoover G , Gilbert S, Curley O, et al Nerve-to-cancer transfer of mitochondria during cancer metastasis. Nature. 2025;644:252-262. 10.1038/s41586-025-09176-8 40562940 PMC12328229

[68] Osswald M , Solecki G, Wick W, Winkler F. A malignant cellular network in gliomas: potential clinical implications. Neuro Oncol. 2016;18:479-485. 10.1093/neuonc/now014 26995789 PMC4799690

[69] Salaud C , Alvarez-Arenas A, Geraldo F, et al Mitochondria transfer from tumor-activated stromal cells (TASC) to primary glioblastoma cells. Biochem Biophys Res Commun. 2020;533:139-147. 10.1016/j.bbrc.2020.08.101 32943183

[70] Civita P , D ML, Pilkington GJ. Pre-Clinical drug testing in 2D and 3D human In vitro models of glioblastoma incorporating Non-Neoplastic astrocytes: Tunneling nano tubules and mitochondrial transfer modulates cell behavior and therapeutic respons. Int J Mol Sci. 2019;20:6017. 10.3390/ijms20236017 31795330 PMC6929151

[71] Nakhle J , Khattar K, Ozkan T, et al Mitochondria transfer from mesenchymal stem cells confers chemoresistance to glioblastoma stem cells through metabolic rewiring. Cancer Res Commun. 2023;3:1041-1056. 10.1158/2767-9764.CRC-23-0144 37377608 PMC10266428

[72] Valdebenito S , Malik S, Luu R, et al Tunneling nanotubes, TNT, communicate glioblastoma with surrounding non-tumor astrocytes to adapt them to hypoxic and metabolic tumor conditions. Sci Rep. 2021;11:14556. 10.1038/s41598-021-93775-8 34267246 PMC8282675

[73] da Silva B , Irving BK, Polson ES, et al Chemically induced neurite-like outgrowth reveals a multicellular network function in patient-derived ­glioblastoma cells. J Cell Sci. 2019;132:jcs228452. 10.1242/jcs.228452 31515278

[74] Sun C , Liu X, Wang B, et al Endocytosis-mediated mitochondrial transplantation: Transferring normal human astrocytic mitochondria into glioma cells rescues aerobic respiration and enhances radiosensitivity. Theranostics. 2019;9:3595-3607. 10.7150/thno.33100 31281500 PMC6587163

[75] Nzigou Mombo B , Gerbal-Chaloin S, Bokus A, et al MitoCeption: Transferring isolated human MSC mitochondria to glioblastoma stem cells. J Vis Exp. 10.3791/55245 2017; PMC5409302 28287607

[76] Guescini M , Genedani S, Stocchi V, Agnati LF. Astrocytes and glioblastoma cells release exosomes carrying mtDNA. J Neural Transm (Vienna). 2010;117:1-4. 10.1007/s00702-009-0288-8 19680595

[77] Babenko VA , Silachev DN, Popkov VA, et al Miro1 enhances mitochondria transfer from multipotent mesenchymal stem cells (MMSC) to neural cells and improves the efficacy of cell recovery. Molecules. 2018;23:687. 10.3390/molecules23030687 29562677 PMC6017474

[78] Oeding SJ , Majstrowicz K, Hu XP, et al Identification of Miro1 and Miro2 as mitochondrial receptors for myosin XIX. J Cell Sci. 2018;131:jcs219469. 10.1242/jcs.219469 30111583

[79] Davis CH , Kim KY, Bushong EA, et al Transcellular degradation of axonal mitochondria. Proc Natl Acad Sci USA. 2014;111:9633-9638. 10.1073/pnas.1404651111 24979790 PMC4084443

[80] Shanmughapriya S , Langford D, Natarajaseenivasan K. Inter and intracellular mitochondrial trafficking in health and disease. Ageing Res Rev. 2020;62:101128. 10.1016/j.arr.2020.101128 32712108 PMC7484258

[81] Caicedo A , Fritz V, Brondello JM, et al MitoCeption as a new tool to assess the effects of mesenchymal stem/stromal cell mitochondria on cancer cell metabolism and function. Sci Rep. 2015;5:9073. 10.1038/srep09073 25766410 PMC4358056

[82] Mulcahy LA , Pink RC, Carter DR. Routes and mechanisms of extracellular vesicle uptake. J Extracell Vesicles. 2014;3 10.3402/jev.v3.24641 PMC4122821 25143819

[83] Liu D , Gao Y, Liu J, et al Intercellular mitochondrial transfer as a means of tissue revitalization. Signal Transduct Target Ther. 2021;6:65. 10.1038/s41392-020-00440-z 33589598 PMC7884415

[84] Lei L , Spradling AC. Mouse oocytes differentiate through organelle enrichment from sister cyst germ cells. Science. 2016;352:95-99. 10.1126/science.aad2156 26917595 PMC6910648

[85] He K , Shi X, Zhang X, et al Long-distance intercellular connectivity between cardiomyocytes and cardiofibroblasts mediated by membrane nanotubes. Cardiovasc Res. 2011;92:39-47. 10.1093/cvr/cvr189 21719573

[86] Jackson MV , Morrison TJ, Doherty DF, et al Mitochondrial transfer via tunneling nanotubes is an important mechanism by which mesenchymal stem cells enhance macrophage phagocytosis in the In vitro and In vivo models of ARDS. Stem Cells. 2016;34:2210-2223. 10.1002/stem.2372 27059413 PMC4982045

[87] Kiriyama Y , Nochi H. Intra- and intercellular quality control mechanisms of mitochondria. Cells. 2017;7:1. 10.3390/cells7010001 29278362 PMC5789274

[88] Davis CH , Marsh-Armstrong N. Discovery and implications of transcellular mitophagy. Autophagy. 2014;10:2383-2384. 10.4161/15548627.2014.981920 25484086 PMC4502649

[89] Wang J , Liu X, Qiu Y, et al Cell adhesion-mediated mitochondria transfer contributes to mesenchymal stem cell-induced chemoresistance on T cell acute lymphoblastic leukemia cells. J Hematol Oncol. 2018;11:11. 10.1186/s13045-018-0554-z 29357914 PMC5778754

[90] Lu J , Zheng X, Li F, et al Tunneling nanotubes promote intercellular mitochondria transfer followed by increased invasiveness in bladder cancer cells. Oncotarget. 2017;8:15539-15552. 10.18632/oncotarget.14695 28107184 PMC5362504

[91] Moschoi R , Imbert V, Nebout M, et al Protective mitochondrial transfer from bone marrow stromal cells to acute myeloid leukemic cells during chemotherapy. Blood. 2016;128:253-264. 10.1182/blood-2015-07-655860 27257182

[92] Bajzikova M , Kovarova J, Coelho AR, et al Reactivation of dihydroorotate dehydrogenase-driven pyrimidine biosynthesis restores tumor growth of respiration-deficient cancer cells. Cell Metab. 2019;29:399-416.e10. 10.1016/j.cmet.2018.10.014. 30449682 PMC7484595

[93] Zhou W , Yao Y, Scott AJ, et al Purine metabolism regulates DNA repair and therapy resistance in glioblastoma. Nat Commun. 2020;11:3811. 10.1038/s41467-020-17512-x 32732914 PMC7393131

[94] McBrayer SK , Mayers JR, DiNatale GJ, et al Transaminase inhibition by 2-Hydroxyglutarate impairs glutamate biosynthesis and redox homeostasis in glioma. Cell. 2018;175:101-116.e25. 10.1016/j.cell.2018.08.038e125. 30220459 PMC6219629

[95] Kidwell CU , Casalini JR, Pradeep S, et al Transferred mitochondria accumulate reactive oxygen species, promoting proliferation. Elife. 2023;12:e85494. 10.7554/eLife.85494 36876914 PMC10042539

[96] Almairac F , Turchi L, Sakakini N, et al ERK-Mediated loss of miR-199a-3p and induction of EGR1 act as a "toggle switch" of GBM cell dedifferentiation into NANOG- and OCT4-Positive cells. Cancer Res. 2020;80:3236-3250. 10.1158/0008-5472.CAN-19-0855 32366479

[97] Spina R , Mills I, Ahmad F, et al DHODH inhibition impedes glioma stem cell proliferation, induces DNA damage, and prolongs survival in orthotopic glioblastoma xenografts. Oncogene. 2022;41:5361-5372. 10.1038/s41388-022-02517-1 36344676 PMC13094125

[98] Shi DD , Savani MR, Levitt MM, et al De novo pyrimidine synthesis is a targetable vulnerability in IDH mutant glioma. Cancer Cell. 2022;40:939-956.e16. 10.1016/j.ccell.2022.07.011e916. 35985343 PMC9515386

[99] Zou Y , Sun Y, Wang Y, et al Cancer cell-mitochondria hybrid membrane coated gboxin loaded nanomedicines for glioblastoma treatment. Nat Commun. 2023;14:4557. 10.1038/s41467-023-40280-3 37507371 PMC10382535

[100] Zhang H , Yu X, Ye J, et al Systematic investigation of mitochondrial transfer between cancer cells and T cells at single-cell resolution. Cancer Cell. 2023;41:1788-1802.e10. 10.1016/j.ccell.2023.09.003e1710. 37816332 PMC10568073

